# Healthcare System Priorities for Successful Integration of Genomics: An Australian Focus

**DOI:** 10.3389/fpubh.2019.00041

**Published:** 2019-03-11

**Authors:** Belinda L. Burns, Gemma A. Bilkey, Emily P. Coles, Faye L. Bowman, John P. Beilby, Nicholas S. Pachter, Gareth Baynam, Hugh J. S. Dawkins, Tarun S. Weeramanthri, Kristen J. Nowak

**Affiliations:** ^1^Office of Population Health Genomics, Public and Aboriginal Health Division, Department of Health, Government of Western Australia, Perth, WA, Australia; ^2^Office of the Chief Health Officer, Public and Aboriginal Health Division, Department of Health, Government of Western Australia, Perth, WA, Australia; ^3^PathWest Laboratory Medicine, Sir Charles Gairdner Hospital, Nedlands, WA, Australia; ^4^Faculty of Health and Medical Sciences, School of Biomedical Sciences, The University of Western Australia, Crawley, WA, Australia; ^5^Genetic Services of Western Australia, King Edward Memorial Hospital, Department of Health, Government of Western Australia, Subiaco, WA, Australia; ^6^Faculty of Health and Medical Sciences, School of Medicine, The University of Western Australia, Crawley, WA, Australia; ^7^Western Australian Register of Developmental Anomalies, Department of Health, King Edward Memorial Hospital, Government of Western Australia, Subiaco, WA, Australia; ^8^Sir Walter Murdoch School of Policy and International Affairs, Murdoch University, Murdoch, WA, Australia; ^9^School of Public Health, Curtin University of Technology, Bentley, WA, Australia; ^10^Faculty of Health and Medical Sciences, School of Population and Global Health, The University of Western Australia, Crawley, WA, Australia; ^11^Harry Perkins Institute of Medical Research, QEII Medical Centre, Nedlands, WA, Australia

**Keywords:** genomics, technology, genetic testing, Australia, healthcare, health system, policy framework, equity

## Abstract

This paper examines key considerations for the successful integration of genomic technologies into healthcare systems. All healthcare systems strive to introduce new technologies that are effective and affordable, but genomics offers particular challenges, given the rapid evolution of the technology. In this context we frame internationally relevant discussion points relating to effective and sustainable implementation of genomic testing within the strategic priority areas of the recently endorsed Australian *National Health Genomics Policy Framework*. The priority areas are services, data, workforce, finances, and person-centred care. In addition, we outline recommendations from a government perspective through the lens of the Australian health system, and argue that resources should be allocated not to just genomic testing alone, but across the five strategic priority areas for full effectiveness.

## Introduction

Genomic testing applications across the human life cycle are continually developing. Genomic testing in healthcare includes the testing of specific genes (technically “genetic testing”) as well as the sequencing of entire genomes and the incorporation of genomic information into disease risk. The use of genomic information to inform healthcare is becoming increasingly common. Associated with these emerging technologies is the potential for growth of prognostic, predictive, diagnostic, and pharmacogenomic testing and screening, which can have relevance at multiple life stages (1). However, access to and governance of these potentially beneficial testing applications varies, with some already being embedded into national, publicly funded health systems while others are offered only in some jurisdictions, only in the private sector or directly to consumers.

Given the advent of genomic testing within these diverse health settings, leadership and coordination is required to ensure the safe and equitable delivery of genomic testing both within and across governmental borders. In the context of the decreasing cost of genomic technologies and their increasing relevance to healthcare, many countries have been restructuring their clinical genetics services to prepare for increasing demand (2–4). International collaboration and communication will be important in order to leverage the lessons learned around sustainable and equitable integration of genomic technology internationally (5). This is particularly true for some of the more universal issues in genomics, such as the availability and implementation of comprehensive and relevant genomic reference databases (5). The successful leveraging of genomic technology to improve healthcare will require a widespread, cohesive and collaborative approach.

Herein, we describe necessary aspects for countries to consider for enabling optimal harnessing of genomic technology for healthcare. We do this by framing discussion around the Australian healthcare system (described in Box 1). Australian governments recently developed the *National Health Genomics Policy Framework* (NHGPF) (10) in recognition of the need for a collaborative approach to the utilisation of genomic technology across the health system. The NHGPF was developed in consultation with the general public and various other stakeholders. The framework was endorsed in November 2017 by the Council of Australian Governments (COAG) Health Council, and delivers a strong and coherent structure from which to coordinate activities across jurisdictions. This framework also represents a shared commitment to implement genomic technology into health systems for the benefit of all Australians. The vision of the NHGPF is “helping people live longer and better through appropriate access to genomic knowledge and technology to prevent, diagnose, treat and monitor disease” (6, p. 5). The mission of the NHGPF is “to harness the health benefits of genomic knowledge and technology into the Australian health system in an efficient, effective, ethical and equitable way to improve individual and population health” (6, p. 5). The NHGPF represents the first national collaboration for health genomics at the whole-of-government level in Australia.

Box 1 **The Australian health system and the funding of health-related genetic and genomic testing.**

The Australian health system is unique. Six State and two Territory Governments, along with the Commonwealth Government are responsible for different aspects of healthcare delivery to citizens, coupled with both public and private healthcare arrangements (6). The Australian health insurance agency, Medicare, provides government funded universal access to healthcare for all Australians. Through this system, specific tests and treatments that have been approved for a Medicare Benefits Schedule (MBS) rebate are provided to patients at no direct cost.

In addition, people can augment their healthcare with privately paid health cover. However, through this private system there is no guarantee that any additional diagnostics and treatments are covered. Health insurance can allow for patients to choose their specialists and healthcare facility, and the agency covers many of these fees. However, insurance companies generally only subsidise genomic tests when a patient is admitted to a hospital, and only if these tests are already covered by the MBS. In 2016, the cost of Medicare-funded genetic and genomic tests was AU$43.5 million, with the value having increased by 24% since 2012 (7). This funding accounts for fewer than 30 genetic and genomic tests, in contrast to the approximately 1,700 such tests that are currently performed by laboratories in Australia (8) at a cost to State or Territory Governments, private healthcare providers, or consumers. Currently, there is no systematic data collection that identifies which tests are funded through these various sources. Perhaps it is due to the extensive process involved in applying for MBS funding for genomic tests and the rapid development of such tests that many are not funded through Commonwealth Government channels. Similar fragmented provision of genomic services occurs in Canada (9).

The NHGPF reflects that for any country to achieve a health system that effectively integrates advances in medical technology such as genomic testing, consideration needs to be given to how to facilitate transparent decision-making, equitable access, provision of a suitable workforce, and effective services that can undergo expansion and redesign (11). This must occur with the support of rigorous assessment of evidence (12) and adequate infrastructure, and in a financially responsible and sustainable manner (10). Successful implementation of expanded genomic medicine services will also take into account the genomic literacy of the whole population, including the nuances of social and cultural norms around the acceptance of genomic information in healthcare (13). These requirements are outlined in the NHGPF's five strategic priority areas of services, data, workforce, financing, and person-centred care.

For countries to ensure universal access and equity of appropriate healthcare are met, an overarching national framework such as the NHGPF for genomics decision-making is necessary. However, governments, as well as local health service providers, must support such a framework to ensure effective, safe and equitable implementation of genomics into health services (3, 10). In this context, we outline some of the key activities for governments based on the five strategic priority areas outlined in the NHGPF, in relation to sustainable integration of genomic testing in healthcare.

## Genomic Services

Government health departments have a responsibility to ensure that genomic tests supplied in their health system are safe and effective for the target population. Genomic testing involves not only a laboratory testing component, but involves associated upstream and downstream services including information provisioning, counselling, interpretation of test results, and clinical decision-making. Implementation of genomic testing into a health system therefore requires consideration of these additional services in alignment with evidence-based best practice. In addition, implementation of genomic testing should be nationally consistent to ensure that all patients have access to the same high quality care. This will require appropriate governance and guidance around safety and quality of services, development of nationally consistent guidance, inter-jurisdictional and international coordination, rigorous processes for assessing the utility of genomic tests, transparent decision-making and timely monitoring and evaluation.

The importance of governance of genomic technology in Australia was recognised more than 15 years ago, when the Australian Government commissioned an inquiry into the use of genomic information. The inquiry was conducted by the Australian Law Reform Commission and the National Health and Medical Research Council (NHMRC). The initial outcome of the inquiry was a report entitled *Essentially Yours: The Protection of Human Genetic Information in Australia* (*Essentially Yours* inquiry) (14). Some of the most relevant recommendations from the inquiry related to the regulatory framework around access to genomic testing, and ensuring privacy and security of genomic information. Changes resulting from the inquiry included amendments to the *Therapeutic Goods Act 1989* to consider all genomic tests, including predictive tests, as *in vitro* diagnostic devices that are regulated by the Therapeutic Goods Administration. These legislative changes ensure that standards around the quality, safety and efficacy of genomic tests are met before supply by pathology laboratories.

Another key recommendation was the formation of a “Human Genetics Commission of Australia.” The government response at the time was to create a principal committee of the NHMRC, namely the Human Genetics Advisory Committee, who were responsible for implementing the recommendations. The Committee developed national guidelines on genomic testing in medical practice (2010), direct to consumer genetic testing (2014) and translating complex “-omics” tests into healthcare, including genomic tests (2015) (15–17). However, the principal committee was ceased in 2015. Functions of the proposed Human Genetics Commission that remain highly relevant for the governance of genomics in the healthcare sector, include (i) providing on-going advice on emerging issues; (ii) development of policy statements and national guidelines; (iii) identifying genetic tests that require special consideration; and (iv) developing practice guidelines for genetic counselling and genetic testing. These functions should be re-considered under the new NHGPF to guide implementation of genomic testing in healthcare, considering the changes in the regulatory landscape that have occurred since the inquiry.

### Governance and Decision-Making Around Genomic Tests

There is a standardised process in Australia for assessing the safety and quality of tests when applying for public funding through the national health insurance scheme, Medicare. For a test to qualify for public funding through the Medicare Benefits Schedule (MBS) an application has to be made to the Medical Services Advisory Committee (MSAC) and the test included on the Australian Register of Therapeutic Goods. The MSAC evaluation process is robust and extensive and involves an assessment of the clinical validity, clinical utility and cost-effectiveness of the test (18). Although many of the safety and quality issues for genomic testing are similar to other types of medical tests, some issues are intensified in the case of genomic testing, and issues may differ depending on the target population or purpose of the test (1). For example, a genomic test may be less effective for population-based screening compared to use as a diagnostic test in a symptomatic individual (1, 19). Therefore, additional guidance and different kinds of evidence may be required around the appropriateness of genomic testing for these and future purposes (20, 21).

Specific evaluation models have been developed for assessing genomic tests, such as the ACCE model developed by the United States of America (USA) Centers for Disease Control and Prevention (CDC) (22). The ACCE model incorporates Analytical validity, Clinical validity, Clinical utility and Ethical, legal and social implications (23). A similar approach was adopted by the United Kingdom (UK) Genetic Testing Network. This concept has also been built on through the CDC's Evaluation of Genomic Applications in Practice and Prevention system for assessing genomic tests. Key learnings from these exercises include the difficulty associated with the heterogeneity of genomic tests, and the importance of defining the purpose of the test. MSAC is currently piloting Clinical Utility Cards to assess genomic tests for predisposition to disease (24). These are based on the Clinical Utility Gene Cards developed by EuroGentest, which in turn were based on the ACCE model.

However, only a small percentage of currently available genetic and genomic tests have been approved so far through the MSAC process, with the remainder funded directly by other parties (see Box 1). If tests are not approved through MSAC, there are a variety of ways that people can still access tests, including direct out of pocket payments, use of health insurance (at the discretion of the insurer), ordering of the tests by clinicians in public facilities with standalone budgets (such as major hospitals) and/or via research programs or clinical trials. The arrangements for these non-MSAC approved tests differ widely across Australian states and territories, are confusing for patients to navigate, and cannot be summarised simply. In Box 1, we provide examples of some existing insurance and payment options to highlight the fragmented nature of the system.

This complex approval process is not unique to Australia; internationally, genomic tests typically take a long time to be incorporated into clinical practice (25). This lag in or lack of approvals for genomic tests may be associated with several factors, including (i) the difficulty in gathering sufficient translational evidence, particularly for tests that only have clinical utility for a small number of patients (21, 26); (ii) the limitations of the indication-specific approval process in the context of rapidly expanding uses for genomic tests (21); (iii) the fact that sometimes a genomic test is not currently required for adequate clinical care; and (iv) the fact that some genomic tests have more personal utility than clinical utility (1, 20). This can be particularly problematic when genomic diagnostic tests are needed to inform novel treatment options for individuals with no existing treatment options or rapid progression of disease (1). Complementary governance frameworks, additional to existing assessment and approval processes, might be necessary to ensure that genomic tests can be evaluated and funded in a timely manner.

#### National and International Coordination and Standardisation

Given the large number of genomic tests that are funded outside of the MBS, coordination and standardisation across jurisdictions is critical for ensuring transparent and equitable decision-making around genomic testing, whilst accounting for local differences in infrastructure. Coordination across states and territories will enable pooling of expertise across jurisdictions, which is particularly important for providing services to people living with rare genetic conditions. Strategic investment into translational research to inform assessment of the benefits and harms of genomic tests for specific population cohorts is required (27–29). In particular, this will help to expand the benefits of genomic technology to different population groups, beyond rare genetic conditions to more common conditions. National networks that identify specific (e.g., gene-, disease-, and/or technology-specific) genomic testing hubs and facilitate coordination of evidence gathering could improve the speed of translation of new genomic tests into clinical practice. Many of these networks already exist in Australia, such as the Australian Genomics Health Alliance (AGHA) and the recently announced Australian Genomic Cancer Medicine Program (30–32). These research collaborations aim to bring together separate parties working toward the same goal, being equitable and effective genomic healthcare for all Australians.

International coordination of genomics policy, particularly in public health, was recognised recently at an international meeting of experts (33). A recent survey of European Union member states revealed that 63% had a policy on genomics in healthcare and 83% of those with a policy had developed specific guidelines (33). In 2018, 13 European countries declared that they will cooperate in cross-border sharing of genomic data, through sharing of infrastructure and expertise (34). A similar international model already exists to help find a diagnosis for people living with rare genetic conditions, the Matchmaker Exchange (35). These international models involve the alignment of policies, data sharing agreements and interoperability of data systems, through federated networks that preserve data governance arrangements for members.

Similar models could be developed across State and Territory Governments to achieve coordinated clinical implementation of genomic testing and ensure equity, sustainability and maximisation of benefits from genomic healthcare initiatives within the public health system. To date, although State and Territory Governments fund many genomic tests, there has been no formal mechanism for governments to strategically coordinate investment to support the implementation of genomic testing in health systems. This means there is an opportunity for further standardisation of decision-making around genomic testing under the NHGPF (36), that works with and complements the existing processes. Consistency in the implementation of genomic testing across the health system is important to ensure all patients receive access to the same high quality healthcare. In Australia this could be achieved through a mechanism to develop standardised policies and/or guidelines aligned with the NHGPF; however this will require further commitment at all levels of government and appropriate engagement with key stakeholders.

In Australia, some other pathology tests are funded by State and Territory Governments, such as the biochemical tests used for the Newborn Bloodspot Screening Programs. Australian jurisdictions have recently developed a *Newborn Bloodspot Screening National Policy Framework* [NBSNPF; (37)], which aligns with the *Australian Population Based Screening Framework* (38), and includes a decision-making framework for the addition or removal of conditions, including specific criteria relating to genetic tests. Similar national standards in relation to the development and implementation of other genomic services and population genetic screening programs may be required in those situations where tests are funded by State or Territory Governments or where different evaluation models are required. Such national guidance was one of the recommendations from the *Essentially Yours* inquiry, although this is yet to be realised. The development of these standards could benefit from learnings from the process undertaken to develop the NBSNPF and the ACCE model.

#### Classification of Genomic Tests to Inform Service Planning and Streamline Governance

A purpose-based classification of genomic tests may help to identify those test types that require specific evaluation approaches, specific expertise (e.g., genetic counselling) or specific upstream or downstream services. Identifying similarities among certain categories of genomic tests may help to streamline the governance and evaluation processes (26, 39). A classification process could also inform the development of national guidelines on what kinds of genomic tests should be provided in a health system and by whom (40). This may include the development of a register of approved uses for genomic tests that can be updated over time and inform guidance for healthcare providers (41). Information on such a register could be utilised to increase the awareness of non-geneticists about genetic testing options and communicate who can order specific tests. This kind of approach has been adopted in the UK through the UK Genetic Testing Network, which promotes equitable access to high quality genomic testing across the UK (42).

#### Horizon Scanning, Monitoring, and Evaluation

A key function of government health departments that could guide the development of genomic policy is the ability to monitor genomic testing usage within health systems. Together with appropriate horizon scanning, the ability to monitor genomic test usage will facilitate the provision of on-going advice on emerging issues. Such a process will help to identify which genomic testing applications are likely to change practice in the immediate and short term to inform key action areas for implementing system-wide change. Although there is a national process for assessing genomic tests for public funding in Australia through the MBS, this process does not involve on-going, routine monitoring to assess the usage and effectiveness of genomic tests. There is also limited evaluation of how genomic services are provided to ensure that healthcare providers comply with agreed standards (16). Close monitoring and guidance around genomic testing is important for ensuring that the necessary infrastructure and workforce is available in the right areas, that genomic services are effective, and over-diagnosis and over-treatment are prevented (12, 43).

Evaluation of genomic testing should involve assessment of population health outcome measures including traditional measures such as reduction of morbidity and mortality, but also impacts on quality of life and reproductive decision-making (20). This function is currently limited in Australia due to a lack of national genomic testing reporting requirements. More robust and transparent data collection on genomic testing activity will allow governments and consumers to monitor and evaluate this part of the healthcare system, to ensure that the use of genomics is safe and equitable, as well as effective. Similar data could also increase our ability to assess the wider benefits of investing in genomic testing, through demonstrating the outcomes of knowledge translation from rare genetic diseases to more common, complex conditions (44). Evaluation is described as a cornerstone for the successful translation of genomics technology into healthcare practice, and is a key function of public health genomics (45–48).

Currently, governments are unable to measure or monitor direct-to-consumer testing usage and are limited in their ability to regulate this activity. Although consumers accessing personal genomic tests may be able to increase their health knowledge and take action to reduce their overall healthcare burden, there is evidence that some direct-to-consumer tests may be inaccurate or misleading and cause undue anxiety (49) or a false sense of complacency (50). There is a need to quantify how many consumers are accessing genomic tests directly through international channels, and determine the impact of this in the Australian regulatory and service planning context. This is likely to be a difficult task, and will require targeted research to survey the usage of direct-to-consumer testing by Australians.

## Genomic Data

### Infrastructure to Support Data Storage and Sharing

The advent of new technologies has enabled rapid, massively parallel DNA sequencing and the production of enormous amounts of genomic data. This has occurred alongside reduced costs of sequencing, decreasing from US$3 billion for the first single genome, to around US$1,000 per genome in 2015 (51). Improved affordability of genomic sequencing enables more widespread accessibility (52), creating an urgent need for adjunct technologies for computation and storage to cope with the expanding demands (53). Until the early 2000s, advances in computation and storage were occurring faster than the ability to sequence DNA and store the respective data. However, with the introduction of massively parallel sequencing, for the first time the demands of genomic informatics out-paced existing models for computation and storage (54).

The cost of sequencing has also been halving every 5 months, much faster than the increases in informatics capacity, placing pressure on the existing genomic informatics ecosystem (54, 55). Genomic testing, particularly massively parallel sequencing, requires substantial computer processing infrastructure as well as bioinformatics expertise to both design the tests and translate raw genomic data into meaningful clinically relevant information (56).

Given the increasing use of massively parallel sequencing in clinical settings, it is likely that increased data storage capacity and developments in data sharing technology will be major enablers for the wider implementation of genomic testing in healthcare. In particular, data storage will be a key consideration for any proposed population-based genomic testing program, particularly any testing that produces a large amount of information (e.g., whole exome or whole genome sequencing). For example, even the data from the 1,000 genomes project in the UK has already reached 200 terabytes in size for just 1,700 genomes (57). Storage of genomic data in Australia is also governed by NPAAC standards, which require storage of certain data files such as interpreted or annotated variant files. Samples may also need to be re-analysed in the short term in order for testing laboratories to comply with the minimum regulatory standards. Recent developments in cloud computing technology are facilitating the collection, use and sharing of large datasets with reduced requirements for expensive data storage infrastructure (58, 59).

Anticipated data requirements will need to be considered for assessment of the minimum infrastructure needs for implementing a genomic test in the clinical setting. The growing application of genomic technology to all aspects of healthcare delivery suggests that the benefits of such technology in improving health are being increasingly recognised. Acknowledging the mismatch between current limitations for capacity of data storage and computation and our improving ability to create large volumes of genomic data, there is a necessity to address these limitations prior to implementation of any genomic testing, particularly at a population-wide level.

### Governance and Privacy of Genomic Data

Apart from infrastructure requirements, the NHGPF also indicates a need for an appropriate level of governance around the collection, safe storage, and sharing of national genomic data (10). Privacy and security of genomic data are important issues, particularly since even a small amount of “de-identified” genomic information can become identifiable, due to the unique nature of an individual's DNA signature. In Australia, genomic information is considered to be sensitive and is protected for private health entities under the Commonwealth's *Privacy Act 1988* (Privacy Act), with each state and territory responsible for the privacy legislation and regulation for public health agencies. For genomic data obtained through research, compliance with the NHMRC *National Statement for Ethical Conduct in Human Research* is required to conduct research projects. Private health entities must adhere to the Australian Privacy Principles contained in Schedule 1 of the Privacy Act, which relates to transparent use, collection, disclosure, quality, security and access to personal information. Entities holding personal information must take reasonable steps to protect information from misuse, unauthorised access, modification or disclosure. There are provisions in the Privacy Act allowing genetic information to be disclosed to family members in circumstances where this disclosure can prevent significant harm to the individual to whom the information relates.

While genetic information is defined as sensitive under the Privacy Act, Australia lacks adequate legislation to protect the privacy of genomic data and prevent genetic discrimination, compared to other countries. In the USA, the *Genetic Information Nondiscrimination Act of 2008* protects the genetic privacy of individuals by preventing insurers from requesting genetic information. Similarly, the European *Oviedo Convention on Human Rights and Biomedicine* (1997), the European Union *General Data Protection Regulation*, and the Canadian *Genetic Non-Discrimination Act* (2017) provide protections for the genetic information of citizens. In contrast, protection against genetic discrimination by life insurers in Australia is self-regulated by the life insurance industry (60). The absence of adequate protections for genomic information has implications for public trust in the collection, storage, and sharing of genomic data by government entities. This in turn may affect research opportunities and precision medicine initiatives enabled through national and international data collection and sharing.

The need for appropriate legislation and mechanisms to support the secure storage of genomic data was highlighted by the recent, controversial introduction of the My Health Record system in Australia. My Health Record is a national electronic health record for all Australians, except those who choose to opt out. This system is capable of storing genomic pathology reports; however, the decision to include genomic data on My Health Record was made without public consultation and seemingly without due consideration of the unique ethical issues pertaining to genomic information, given that this information is heritable in nature (61). Questions have arisen around the security of the system, as well as the ability of government agencies to access health records, and have raised concerns among health professionals and the public. A national, population-based electronic health record has enormous potential for furthering genomic research efforts. However, equally, a lack of transparency and appropriate consultation could permanently damage public trust and participation in the system. Therefore, further consideration is required around ethical issues and appropriate safeguards, as well as robust public consultation, before genomic data is uploaded onto the My Health Record system.

## Genomics Healthcare Workforce

The current skillset required to deliver genomic healthcare is broad and varied depending on the application of testing. Generally, some or all of the following professionals may be involved: laboratory scientists, clinical pathologists, bioinformaticians, clinical geneticists, genetic counsellors, and non-genetics healthcare professionals. The genomics healthcare workforce must have adequate genomics literacy to know when to order genomic tests; how to interpret genomic tests to inform clinical decision-making; how to counsel patients on genetic conditions and genomic tests; how to obtain informed consent before a test or procedure; and how to ensure understanding and appropriate action following a test result or procedure (62). These aspects of genomic clinical expertise can be broadly categorised into two distinct domains: (i) clinical gatekeeping (ordering and interpreting genomic tests, including clinical utility), and (ii) counselling and consent.

Generally, genomic tests warrant a greater level of expertise than other medical tests and the provision of professional genetic counselling around medical decision-making, including reproductive options, due to the uncertain outcomes of testing and implications for genetic relatives. At the very least, the use of genomic tests requires a medical workforce that is confident undertaking appropriate genomic risk assessments and communicating this information to patients (62). However, the type of model for gatekeeping genomic testing, genetic counselling and seeking consent will depend on the characteristics of the condition/s being tested for and the test.

As was previously noted, the ability of Australian governments to predict demand for certain types of genomic tests is currently limited due to the lack of a national monitoring program. Nevertheless, there is evidence that the current model for ordering, interpreting and providing counselling around genomic tests is not feasible even if there was national agreement on their criteria (2, 63). This has been recognised for some time; for example, the *Essentially Yours* inquiry recommended that Australian governments “develop strategies to assess and respond to the need for increased and adequately resourced genetic counselling services” (14) and examine options for development of genetic counselling as a profession.

Training options are limited for potential counsellors in many Australian jurisdictions. Currently, genetic counselling is self-regulated, with counsellors choosing to become certified by the Human Genetics Society of Australasia (HGSA). In 2017, a working group of the HGSA was formed to explore the issue of regulation for this profession (64). A submission is being prepared to have genetic counselling professionally regulated through the National Alliance of Self Regulating Health Professions, which will facilitate consistency in practice and ensure quality in services.

Anecdotally, there is increasing demand internationally for complex genomic tests, particularly as part of population-wide screening programs (65, 66) and expansion beyond the diagnostic use of genomic testing to include screening and other uses (67). This is already putting pressure on expert genetic workforces (39, 68). In addition, with increasing use of somatic genomic testing in oncology, it is possible that more patients with germline mutations could be identified, requiring attention from clinical genetics centres (69, 70). In this context, how can we prepare the workforce for an inevitable increase in the use of genetic information in managing patient care? It may be necessary to reconsider current best practice approaches to delivering such care, by deconstructing the workforce requirements relating broadly to each type of genomic test. This may require a multi-pronged approach across the whole health system.

### Clinical Gatekeeping

One approach to ensuring availability of an appropriate genomics workforce is to mainstream genomics education into the core of medical education (62). Online learning tools may help to increase access to genomic education and facilitate sharing of efforts nationally and internationally (5). Without appropriate education, there is a limited ability for non-geneticist specialists to know which patients could benefit from more complex genomic testing (12, 62, 71).

Alternative scenarios include general practitioners, specialist (non-geneticist) clinicians and even pharmacists directly ordering genomic tests from pathology providers to inform the clinical management of their patients (72–75). Nevertheless, genetic specialists have expressed concerns over the ability of non-genetic clinicians to order particular genomic tests, due to a potential lack of knowledge and understanding required for informed consent and reporting of results (75). Additionally, some genomic tests offered by international laboratories are available direct to consumers, often bypassing healthcare professionals as gatekeepers for decision-making (76). In certain cases, these companies require a referral or review of results by a doctor, but this may be a company employed doctor rather than an individual's personal doctor. A consolidated list of agreed uses for genomic testing in general practice or specialist settings could facilitate the mainstreaming of genomic tests, combined with the embedding of this guidance into workflows, such as the Health Pathways being developed by Australian primary health networks.

Certain uses for genomic testing may be more amenable to mainstreaming when compared to others. For example, once clinical utility can be agreed upon, it may be appropriate for non-genetics healthcare professionals to order certain tests, such as pre-conception, prenatal, and diagnostic tests and cascade screening tests for common conditions. This is already occurring with mainstreaming of hereditary ovarian and breast cancer testing (69, 77), and in Australia, tests for these conditions have recently been made available to be ordered by any healthcare practitioner under the MBS (78). Similar mainstreaming has occurred with many (non-genomic) medical tests, even to the point of direct consumer access (e.g., HIV testing) (79), although this remains controversial in many cases (80). However, mainstreaming of medical device use will take time, as adequate training of relevant members of the workforce will be required to ensure genomic testing occurs in a safe and appropriate way. For example, the Public Health Genomics Foundation in the UK has recommended the establishment of core competencies for ordering genomic testing (81).

In other instances, the involvement of genetic specialists may not be easily replaced. For example, clinical geneticists, clinical pathologists and bioinformaticians will continue to be required for testing associated with complex rare diseases and where whole exome or whole genome sequencing is being used with less targeted filtering applied for analysis. Given the dependence of implementing genomic technology on computer-based interpretation of sequence data, there is a need to ensure a suitably qualified bioinformatics workforce is in place to enable translation of this information into clinically meaningful results (47). Specific expertise is required to accurately interpret the information in a way that can inform clinical action to ensure the utility of genomic information in the clinical setting. Within Australia and internationally, there are examples of recognised super specialties and joint residencies and fellowships in genetics for paediatric, maternal-fetal and internal medicine specialties, and for cancer genetics and neurogenetics (62, 82). These all acknowledge the need for genomics expertise in particular settings.

Additional models for the gatekeeping of genomic testing exist, and these may need to be implemented into local programs (63). Examples include the increasing relevance of multi-disciplinary and interdisciplinary clinics, and coordination among health professionals in the diagnosis and management of patients with or at risk of genetic conditions (2, 4, 71). Multi-disciplinary teams have already been used as part of best practice for areas such as cancer genetics and rare genetic diseases (2), and are also being used in particular specialties such as cardiac, renal, liver, lung, and neurology clinics. However, particularly with gatekeeping around massively parallel sequencing technologies applied to whole exome or whole genome sequencing, and with increased demand for predictive and pre-symptomatic testing, multi-disciplinary teams may become increasingly necessary in other settings to facilitate reporting results back to individuals (3, 71, 75).

### Counselling and Consent

Generally, genetic counselling is offered where individuals or their offspring are identified as being at higher risk than the general population of developing a genetic condition from a genetic or non-genetic (e.g., cholesterol) screening test, or due to family history. Traditionally the scope of practice has been focused on supporting people with certain Mendelian-inherited genetic conditions. According to the Australasian Society of Genetic Counsellors, genetic counselling is “a communication process, which aims to help individuals, couples and families understand and adapt to the medical, psychological, familial and reproductive implications of the genetic contribution to specific health conditions.” Specific functions of a genetic counsellor include assessing risk, educating patients and families about a genetic condition, providing guidance around decision-making, and facilitating adjustment after a new diagnosis.

Depending on the condition, genetic counselling is usually offered close to the time that any complex decision-making is to occur, such as the decision to have children, undergo pre-implantation genetic diagnosis, undergo invasive diagnostic testing, or terminate a pregnancy when high risk for a condition is identified or following confirmation of a diagnosis. Many professional bodies consider the provisioning of professional genetic counselling with all genomic tests best practice. However, in reality if there is a substantial benefit to offering a genomic test to a larger population, this will not be feasible to implement and alternative models will be required where they are deemed appropriate based on a risk assessment. A key area for the need to find alternative models for delivery of genetic counselling is in any application of population-wide genomic testing. For example, there is growing interest in introducing reproductive genetic carrier screening for certain rare inherited conditions where there may be no family history (e.g., recessive and X-linked conditions) (83–85).

Lessons could be learned from similar programs or practices such as cancer genetic counselling where demand for testing has begun to outstrip the available supply of genetic counsellors. For example, evidence from the USA has indicated that the majority of women tested for *BRCA1* and *BRCA2* variants are not receiving any genetic counselling (86). Alternative models could involve mainstreaming genetic counselling among non-genetics experts, such as genetic counselling provided by practice nurses with a special interest in genetics (4, 87, 88), counselling by the general practitioner or specialist, expanding the practice of genetic counsellors outside specialist genetics centres (4), or offering online counselling.

Preliminary evidence suggests that online delivery of information and counselling for carrier screening is equivalent to or non-inferior to in-person genetic counselling (89–91). Such alternative models are considered acceptable by some peak bodies in the USA (92). However, this approach has not been robustly tested among a pre-conception population or among populations with lower genomic literacy compared to research study participants. A further alternative model for providing pre-test genetic counselling and obtaining informed consent in the context of increasing demand involves group counselling. This model of service delivery has already been used in the prenatal and cancer genetics settings (86, 87, 93, 94). Finally, telehealth genetic counselling has been utilised extensively in cancer genetic counselling (95), and in other settings such as prenatal counselling (96). Recent developments in this space include the incorporation of chatbots (artificial intelligences with ability to converse via textual or auditory mediums) to help triage patients (97).

## Financing Genomics

Like other countries globally, Australia's health expenditure is increasing faster than the inflation rate, and in a climate of budgetary constraints, there is a necessity for greater accountability in health expenditure to create truly sustainable public healthcare systems (98). For example, the present state of Australia's health systems is exemplified by Australia's 2015–2016 ratio of health expenditure to GDP at 10.30%, up from 8.68% in 2005–2006 (99). With fragmentation of healthcare across public, private, as well as state and commonwealth systems, the funding arrangements for genomic testing vary for different applications throughout the life cycle, as well as by jurisdiction.

In the context of the potential benefits of genomic technology, governments should be investing into the basic infrastructure and workforce required to support genomic healthcare and should invest in clinical DNA sequencing, data storage and computation infrastructure. Much of the investment in other countries so far has focused on funding large-scale research efforts, such as the 100,000 genomes project in the UK, the All of Us precision medicine research program in the USA, the Pilot Program for Personal Medicine in Estonia, Genome Canada's National Precision Medicine Initiative, and similar projects in China, Saudi Arabia, Dubai, and Turkey. Investments like these have involved the building of the capability for genomic sequencing (5).

The UK and USA are ahead of most other countries in beginning to translate the results of this research investment into the healthcare system. Both countries have had dedicated public health genomics centres since 1997. In June 2018, a United States Senate Appropriations Subcommittee approved an US$86 million increase for the All of Us precision medicine research program, which now operates with a budget of US$376 million from the National Institutes of Health. In the UK, part of the 100,000 genomes project included £20 million over 4 years for a Genomics Education Programme (3), and in 2018, the National Health Service in the UK started offering whole genome sequencing routinely for patients with rare diseases and certain cancers (100).

In Australia, there are a number of recent genomics research initiatives at a state, territory and national level. Several state-wide collaborative research entities have been developed aimed at harnessing healthcare, industry, and research expertise to determine how genomic testing can be incorporated into routine clinical practice (101). Similarly, the Australian Genomics Health Alliance, a research project funded by the NHMRC, aims to understand and address challenges associated with integrating genomic medicine into Australian health systems. Genomic testing is currently on the Commonwealth Government healthcare agenda, as evidenced by the allocation of AU$500 million in research funding to the Australian Genomics Health Futures Mission (102).

Along with these research efforts, health system implementation of genomic testing has also occurred in recent years in Australia. For example the Western Australian (WA) Government has a dedicated Office of Population Health Genomics. This Office has facilitated the alignment of existing resources within the WA health system and has developed policy to support a rare and undiagnosed diseases diagnostic service (103). An impact analysis of the service demonstrated a three-fold increase in confirmed diagnostic outcomes for the WA population. Similarly, the Victorian State Government provided AU$8.3M for the 2017/2018 financial year to enable publicly funded genomic sequencing for individuals with rare diseases, along with associated genetic counselling and multidisciplinary clinical care. Initial phases of the genomic test implementation were reported to be delivering six times the number of disease diagnoses compared to the previously available tests, at a quarter of the price (104).

Australia needs further investment in embedding genomics expertise into commonwealth and state health departments and health services to ensure appropriate oversight and strategic benefits realisation associated with genomic healthcare. Key priorities for government funding in the genomic healthcare space might include investing in a robust monitoring and evaluation system, ensuring that appropriate sequencing and data infrastructure is available to support increased demand, improving reimbursement/funding streams for multi-disciplinary teams, and assessing cost-effectiveness of population based genomic screening programs.

Participation in multi-disciplinary and inter-disciplinary meetings is currently not adequately funded by the Commonwealth Government's activity-based funding for hospitals. Improving the funding pathway for this activity will be important for ensuring that genomic testing is utilised for those patients who need it most and that the appropriate clinical guidance is available. Processes are underway to improve this situation, such as reviewing current funding mechanisms to better reflect workloads (105). Similarly, ensuring that there is adequate reimbursement that recognises the necessary clinician time for interpretation of test results in preparation for appointments may be important (71).

### Strategic Prioritisation of Investment Across Different Healthcare Settings

In a public healthcare system with finite resources, prioritisation of services is necessary (36). Agreed and consistent qualifiers to determine prioritisation can inform allocation of resources. One suggested approach for prioritising genomic tests is to favour those who are at high risk of imminent, serious, preventable conditions that are cost effective to treat (106). Other factors to consider are the severity of disease impact, the availability of prevention or a targeted treatment, and acceptance of the net cost for health gains achieved (36, 106).

Individual review of genomic tests, while necessary to establish analytical and clinical validity, clinical utility and cost-effectiveness, is onerous and resource intensive (36, 41). As a result, the individual assessment of tests contributes to the *ad hoc* approach to funding genomic tests in healthcare systems (9). This is particularly the case for applications used in the context of rare diseases and lethal conditions where limited baseline evidence is available to inform adequate review or where time is limited due to the progression and severity of the disease (27).

Part of the prioritisation process involves considering the opportunity costs of investing in one healthcare service over another, equally important service. Individual assessment methods based on medical need do not always take into account any comparison with investment into other services. Higher level considerations for prioritisation of healthcare services include maximising health gains for the population and addressing inequities in access (36). Considering the diverse criteria contributing to the need for genomic testing and the need for a more strategic approach, a potential area of interest is the development of a multi-criteria decision-making framework, such as that developed by the UK Genetic Testing Network (41). This framework involves ranking of genomic tests by a group of representative stakeholders, according to weighted criteria relating to reducing morbidity and mortality, enhancing reproductive choice, improving the process of care, deliverability of services and additional information (41). A similar model could be useful in Australia.

### Cost Savings

In Australia, multiple funding arrangements exist for various genomic testing purposes creating issues for equitable access. Given the inevitable increasing use of genomic testing in the healthcare system, it is possible that a large proportion of the population will eventually have at least one genomic test during their lifetime. A cohesive approach to funding and access for genomic testing may ultimately provide cost savings to society. For example, for particular cohorts such as those with rare diseases, there is the potential for whole exome, whole genome, or targeted gene panel sequencing to produce cost savings by avoiding a long series of other genetic tests (3). A recent Australian study that investigated the health economic impact of whole exome sequencing for infants with a suspected monogenic disorder found a cost saving of AU$1,578 per quality-adjusted life year gained at 1 year, revealing an overall cost-benefit to the health system when genomic testing is incorporated into clinical care for this subset of patients (107). Learnings from rare diseases may also inform more targeted approaches to treatment for common, complex conditions, which could translate the benefits of reduced morbidity and mortality to the population at large, thereby increasing the cost savings of the initial investment (44).

In contrast, the use of genomic information may lead to increased need for healthcare services in the short term (108), such as genomic testing indicating an increased cancer susceptibility that encourages earlier and/or more frequent screening and heightened vigilance that would not have occurred in the absence of the genomic test. However, these costs may be offset by the savings from detecting cancer early, thereby avoiding deterioration in the patient's condition and possibly providing a better prognosis. This will of course depend on the availability and effectiveness of interventions and treatments, and if test results translate to behavioural change. Likewise, the application of genomic testing for complex polygenic diseases may be more cost effective if it is able to identify specific treatment options that are more likely to be efficacious (106) and be used. An outcomes-based approach to monitoring and evaluation will help to inform timely and strategic funding decisions.

## Person-centred Care

As with any new medical technology, the successful integration of genomic medicine into healthcare delivery will rely not only on workforce engagement in the new technology, but also on the engagement of and acceptance by the greater population. Adequate understanding of genomic testing by the general public is required in order to obtain truly informed consent from patients. Future genomic medicine initiatives will need to be delivered in a way that is sensitive to the ethical, legal, and social issues associated with genomic information.

### Genomic Literacy in Healthcare Consumers

Addressing public engagement and literacy in genomics is even more important in the context of the increasing availability of direct-to-consumer testing, although a recent study suggested that Australian consumers' awareness of such tests is not as high as would be expected based on media reports (109). Similarly, recent surveys in the USA and UK on public opinion on personalised medicine and genomics found that most respondents were not familiar with these concepts (3, 110).

Successful engagement of the general public will require public health education and promotion programs that consider the nuances of public health behaviour change, such as the utilisation of behavioural economics (111). This approach acknowledges that new policies and technology alone are unlikely to catalyse changes in health behaviours. Significant learnings can also be drawn from the use of deliberative public engagement methods, which have been used to explore community opinions on similarly complex issues like biobanks (112, 113) and personalised medicine (114). Aspects to consider that ultimately affect an individual's choice to engage in a health service are strong fear of loss, considerations of the social norm, and emotional associations (111, 115).

There are also additional implications of obtaining greater genomic knowledge, such as the potential for perceived stigma associated with knowing carrier status (13), psychosocial impacts (116), negative effects on family dynamics (116), and privacy concerns such as fear of limitations on access to insurance (109, 116). The variable perspectives on the utility of genomic testing should also be deliberated when designing any public education and engagement interventions. Consideration should be given to providing educational interventions that are culturally appropriate, including language-appropriate communication materials (117).

Individuals in the community are likely to perceive information from governments and independent academic agencies to be legitimate (109). Consequently, appropriate information produced by these organisations, such as the NHMRC's resources for consumers, should be utilised to educate the general public and build acceptance in the community about genomics in healthcare. Strong leadership from governmental health departments will be critical to the success of raising public acceptance of genomic healthcare (48, 118).

### Equity of Access to Genomic Tests and Their Health Benefits

The lack of existing national, state and territory policies and procedures in Australia surrounding access to genomic testing can lead to inequitable access. At the outset, effort should be made to ensure culturally appropriate genomic services are available for all. This includes minimising any disparity due to where people live, particularly those living in rural and remote areas, through informed service planning and telehealth solutions. The so called “post-code lottery” could currently result in differences in which genomic tests are offered to individuals, if indeed any are suggested or offered at all. Other possible areas of inequity include a lack of appropriate reference genomes (1, 119, 120); difficulty accessing international clinical trials for people with rare diseases; variation in access to publicly subsidised treatment options based on traditional cancer classifications; inequity of access to genomic tests based on ability to pay for tests that are only available in the private sector; and the potential for individuals with a higher education, genomic literacy and/or financial means to more readily access direct-to-consumer tests.

For example, currently in Australia pre-implantation genetic diagnosis (PGD) is not publicly funded. As such, this represents an inequity in enhanced reproductive choice for couples at higher than usual risk of having an offspring with a genetic condition who may wish to access such a technology to proactively prevent their future child having or developing that specific genetic disease/s. MSAC initially supported PGD to be publicly funded, however on further consideration advised that it was not appropriate for MBS listing, partly due to likely costs being largely speculative and complexities in implementation, and requested further information be gathered (121). Certainly providing PGD under a publicly funded scheme would achieve the NHGPF's goal of providing national consistency for equity.

Like PGD, private payment for non-invasive prenatal screening (NIPS) poses problems for equity of access when attempting to incorporate NIPS as part of widespread uptake into routine antenatal care. It has been estimated that with advances in technology, the cost per NIPS test will fall under AU$500 in the near future. However, as with PGD, any incorporation into routine antenatal care will require a stringent economic analysis for benefit (122) and cost utility such that efficient and transparent allocation of public resources can be achieved. NIPS is considered to have superior rates of detection compared to traditional prenatal screening methods for chromosomal abnormalities due to improved sensitivity and specificity. This means that fewer invasive diagnostic procedures are subsequently required, resulting in lower rates of procedure-related miscarriage (123). However, current prenatal screening programs that utilise ultrasound services are able to detect structural abnormalities of the foetus that would not be identified through NIPS, thus it is unlikely that NIPS alone will supersede all facets of the current prenatal screening program.

Serious consideration should be given to the infrastructure required to ensure that genomic testing is equitably accessible to all, and that there is culturally safe, timely and optimised outcomes and benefits. Focused effort is required to ensure that genomic tests are appropriate and accessible to disadvantaged groups and underserved populations (117). This will involve targeted research and significant stakeholder engagement to improve translation of genomic testing to benefit all members of the population. This effort extends to ensuring that genetic counsellors and other members of the healthcare workforce providing counselling to patients have the opportunity to engage in cultural sensitivity training. Other key priority areas for governments include public education campaigns, developing patient decision aids, integrating genomics into health promotion and disease prevention programs, empowering local community groups, and involving consumers in policy development. Moreover, incorporation of patient-facing interfaces in electronic medical records that contain genomic information and are accessible to patients will help to close the loop and ensure that patients feel involved in their healthcare (124).

## Conclusions

A strategic, holistic, and cooperative inter-governmental approach is needed to enable the successful integration of genomic testing into existing healthcare systems. Such an approach will help to prevent process duplication while also standardising genomic test implementation across jurisdictions, ensuring equity of access for a range of test applications, and identifying cost-savings through shared infrastructure and strategic planning.

The NHGPF in Australia serves as a guide, signposting areas for consideration prior to the implementation of a nationwide genomic testing strategy and directing key points for discussion for the purposes of this review. Successful implementation of the strategy is likely to require on-going leadership and coordination around genomic healthcare from governments and prioritisation of key healthcare settings for implementation.

The financial impact of expanding the use of genomic testing must be considered within the context of the NHGPF strategic goals for ensuring the sustainability of health service delivery, while simultaneously overcoming inequities of access, and delivering person-centred care. All stakeholders including the patient/individual and their family, clinicians, genomic technology companies, geneticists, molecular pathologists, laboratory scientists, bioinformaticians, and policymakers should be brought together as partners to help decide the future of genomic healthcare. However, a certain degree of genomic literacy is required by everyone who will be involved in such discussions, to facilitate significant engagement and shared decisions about the application of genomic tests and interpretation of results.

## Author Contributions

KN, BB, GAB, and HD conceived the paper concept. BB, GAB, EC, and KN drafted the manuscript. All authors provided critical input and approved the final manuscript version for submission.

### Conflict of Interest Statement

The authors declare that the research was conducted in the absence of any commercial or financial relationships that could be construed as a potential conflict of interest.
